# Role and Importance of IGF-1 in Traumatic Brain Injuries

**DOI:** 10.1155/2015/736104

**Published:** 2015-08-31

**Authors:** Annunziato Mangiola, Vera Vigo, Carmelo Anile, Pasquale De Bonis, Giammaria Marziali, Giorgio Lofrese

**Affiliations:** ^1^Institute of Neurosurgery, Catholic University School of Medicine, Largo Agostino Gemelli 8, 00168 Rome, Italy; ^2^Sant'Anna Hospital, Via Aldo Moro 8, 44100 Ferrara, Italy

## Abstract

It is increasingly affirmed that most of the long-term consequences of TBI are due to molecular and cellular changes occurring during the acute phase of the injury and which may, afterwards, persist or progress. Understanding how to prevent secondary damage and improve outcome in trauma patients, has been always a target of scientific interest. Plans of studies focused their attention on the posttraumatic neuroendocrine dysfunction in order to achieve a correlation between hormone blood level and TBI outcomes. The somatotropic axis (GH and IGF-1) seems to be the most affected, with different alterations between the acute and late phases. IGF-1 plays an important role in brain growth and development, and it is related to repair responses to damage for both the central and peripheral nervous system. The IGF-1 blood levels result prone to decrease during both the early and late phases after TBI. Despite this, experimental studies on animals have shown that the CNS responds to the injury upregulating the expression of IGF-1; thus it appears to be related to the secondary mechanisms of response to posttraumatic damage. We review the mechanisms involving IGF-1 in TBI, analyzing how its expression and metabolism may affect prognosis and outcome in head trauma patients.

## 1. Introduction

Biomarkers are indicators of a specific biological or disease state that can be measured in both the affected tissue and peripheral body fluids. These markers are represented by altered enzymatic activity, changes in protein expression or posttranslational modification, altered gene expression, protein or lipid metabolites, or a combination of these parameters [1]. Over the years an increasing importance has been placed on the analysis of disease-specific biomarkers, thus revolutionizing the diagnostic, prognostic, and therapeutic approach of various human pathologies [2], including cancer, heart failure, infections, genetic disorders, and traumatic injuries [1]. On these grounds, in the last years a growing interest has developed in biochemical markers of brain damage related to traumatic and vascular events [3].

Traumatic brain injury (TBI) is a nondegenerative, noncongenital insult to the brain from an external mechanical force, causing temporary or permanent neurological dysfunction. It is a common cause of death and disability in industrialized countries for both adults and children, with sequels ranging from physical disabilities to long-term behavioural, cognitive, psychological, and social defects [4]. Under the TBI the injury has to be distinguished primarily, caused by the mechanical damage to the nervous and vascular structures, and secondly, due to the evolution of a cascade of secondary events that compromise the function, structure damage and further promote cell death [5].

The neurological insult and outcome of TBI patients are both currently diagnosed and estimated through clinical examinations of the level of consciousness such as Glasgow Coma Scale; various imaging techniques, including CT, MRI, and positron emission tomography; and assessment of other vital parameters (e.g., intracranial pressure and electroencephalogram) [6]. These diagnostic tools have proved to be frustratingly limited, especially in the intensive care unit setting; thus the search for surrogate markers, detectable in serum and/or CSF, could provide further information about the extent of neuronal damage, which is crucial in estimating prognosis and outcome [6].

Different studies have proven that most of the long-term consequences of TBI are due to molecular and cellular changes occurring during the acute phase of the injury and which may, afterwards, persist or progress [7, 8]. Because of these reasons, the search for predictive serological markers of outcome in TBI began over 20 years ago [9], and the list of putative biomarkers for traumatic brain injury continues to grow as do the conflicting results of their utility in various injury paradigms [10]. A variety of proteins, small molecules, and lipid products have been proposed as potential biomarkers of brain damage from TBI [1].

To date, the majority of TBI researches have been focused on protein profiling such as S100B, GFAP, NSE, MBP, FABPS, a-II spectrin, phosphorylated neurofilament H, and ubiquitin C-terminal hydrolase, which can be all identified in serum or/and cerebral spinal fluid (CSF) helping to evaluate injury severity and correlate with morbidity and mortality [1].

Another important modification observed after TBI is the increased serum and/or CSF concentrations of acute phase proteins (e.g., C-reactive protein, amyloid A, proinflammatory cytokines (e.g., IL-1, TNF-a, and IL-6), anti-inflammatory cytokines (IL-10, transforming growth factor beta, or TGF-b), and chemokines (e.g., ICAM-1, macrophage inflammatory protein- (MIP-) 1, and MIP-2)). The CSF and/or serum level modifications of these markers have been related with injury and sometimes with outcome through time-specific changes in response to TBI [1].

Among the potential biomarkers involved in primary and secondary injuries should be counted also metabolites of neurotransmitters, second messengers, ions and glycolytic intermediates, such as cAMP, whose concentration in CSF was found to correlate with the grade of coma, or N-acetylaspartate (NAA) that seems to predict eventual neuropsychological deficits [1].

During the last two decades many evidences have suggested a hormonal crucial role in influencing the damage after TBI, being hormones usually involved in the stress response occurring in critical illness [11]. Therefore several studies focused their attention on posttraumatic endocrine dysfunction, attempting to correlate it with TBI outcome. In this contest, blood modifications of growth hormone (GH) and IGF-1 concentration appear to be the most affected, with various authors increasingly assigning a greater value to IGF-1. This molecule seems to play important roles in both the pathogenesis and the secondary response to brain damage. Thus we tried to understand, through the literature, if there are grounds to identify the IGF-1 as a crucial marker in serum and CSF of those patients suffering from traumatic brain injuries.

## 2. IGF-1 in the CNS

The IGF-related peptides may affect brain function by either local tissue expression or by peripheral circulating peptides crossing the BBB via transcytosis [12]. IGF-1 is part of a well-structured family peptide. The IGF signaling system is composed of three ligands (IGF-1, IGF-2, and insulin), three cell membrane receptors (IGF-1R, IGF-2R, and the insulin receptor IR), and several associated proteins, namely, IRS and SHC. IGF-1 circulates in the plasma as complexes formed with IGFBPs that probably serve several biological functions. The vast majority of IGF-1 (99%) is bound to IGFBP3 or IGFBP5 and is coupled with a glycoprotein called the acid labile subunit. The final binding of IGF-1 to its own receptor IGF-1R triggers a conformational change that causes tyrosine autophosphorylation and transphosphorylation, enhancing its tyrosine kinase activity [13]. These events bring about recruitment of IRS, CRK, and SHC, leading to the activation of three main pathways: the MAPK/Ras-Raf-Erk pathway, the phosphatidylinositol-3-kinase/AKT/mTOR (PI3K/AKT) pathway, and the Janus kinase/signal transducer and activator of transcription (JAK/STAT) pathway [14]. There are two sources of production of IGF-1, yielding different functions to this molecule: the liver generates IGF-1, which acts as a prolongation of the GH under tonic pituitary stimulation of hepatic synthesis; IGF-1 is also produced locally by many types of peripheral cells under basal conditions and in response to inflammatory stimuli. In this sense, although it is mainly produced by the liver (70%), IGF-1 can be secreted by every tissue. More specifically IGF-1 and the IGF-1R are expressed in close proximity to each other in various brain regions, suggesting a paracrine or autocrine functional loop in physiological and pathophysiological mechanisms [15]. Receptors for IGF-1 are virtually present on all cell types but they are mainly located on those cells of mesenchymal origin, such as fibroblasts, chondrocytes, and osteoblasts [16]. In human's brain, IGF-1 receptors are found predominantly in the hippocampus and parahippocampal areas, but also amygdala, cerebellum, and cortex express them [17]. BBB uptake of circulating IGFs involves the IGF-1R and the low-density lipoprotein receptor-related protein 1 (LRP1), through which IGFs can reach the CSF as well as the aforementioned anatomical targets [12]. Although there is evidence that IGF-1 is transported across the BBB via transcytosis [18], a significant amount of IGF-1 is undoubtedly produced in the brain, confirmed by the fact that IGF-1 mRNA has been found predominantly in the adult rats brain stem and cerebellum [19].

IGF-1 stimulates the proliferation and differentiation of oligodendrocytes supporting myelination of the CNS, being involved in the differentiation of neurons to specific cell types. It can increase levels of neurotransmitters, neurotransmitter receptors, and proteins of the cytoskeleton; it can inhibit apoptosis in neurons [19]; it stimulates dendrite growth, angiogenesis, and amyloid clearance [12, 20].

Moreover disruption of the IGF-1 gene, leading to loss of function, induces neuronal loss in the hippocampus and striatum [21]. As demonstrated in aged rats, there is a permanent neurogenesis in the dentate gyrus of the hippocampus of mammals decreasing with age up to a 60% reduction in the differentiation of new cells to neurons. This trend depends on environmental factors, hormones, and growth factors such as IGF-1 and this evidence is confirmed by the fact that reduction could be reversed by intracerebroventricular administration of IGF-1 [22]. Thus, it can be assumed that the age-dependent decline in the expression of both IGF-1 and IGF-1 receptor could be a possible contributing factor to the development of cognitive deficits seen in the elderly.

These cognitive impairments were reversible by prolonged systemic administration of IGF-1 and suggested that the neurotrophic actions of IGF-1 affect glutamatergic synapses within the hippocampal circuitries, thereby affecting learning and memory [12].

## 3. IGF-1 in the CNS Pathologies

IGF-1 plays an important role in brain growth and development [23], and it is involved in repair responses to damage for both the central and peripheral nervous system [24–26]. IGF neurotrophic activity, together with its binding proteins and signalling receptors, is suggested to be fundamental in the recovery of neural tissue from injury [27]. This evidence is supported by the CNS response to injury through the upregulation of the IGF-1 expression.

In this sense different studies concerning the CNS have revealed an impressive IGF-1 induction after different brain insults such as ischemia [28, 29] and cortical injuries [30–32] as well as injuries of the spinal cord [33]. The major role of IGF-1 in hypoxic/ischemic damage, through its modulation of the cellular response stimulating the repair mechanisms, is increasingly being recognized. Serum IGF-1 levels have been proved to be depressed following acute stroke in the human being [29, 34], while in rodent models brain IGF-1 levels resulted in increase in the perilesional stroke area [28], thus likely revealing a neuroprotective role. It seems also that poststroke serum IGF-1 levels are correlated with outcome from ischemic brain injury, with its higher levels reducing lethality [34]. In the wake of this evidence many studies have shown the beneficial effect of IGF-1 administration after stroke, reducing neuronal loss and infarct volume, while increasing glial proliferation [35, 36].

A significant body of data has identified IGF-1 both as a major regulator of amyloid *β*-peptide (A*β*) physiology and as an important factor in the pathogenesis of Alzheimer's disease (AD) [12]. A recent study demonstrates that lower IGF-1 serum levels are associated with an increased risk of developing AD dementia, while higher serum results are related to greater total brain volumes and may protect against subclinical and clinical neurodegeneration [37].

Moreover, IGF-1 appears to be linked with repair processes after brain damage, controlling the regeneration of injured peripheral nerves [38] seeming to be relevant in ameliorating clinical outcomes in animal models of amyotrophic lateral sclerosis [38]. Some data also suggest that aberrations in IGF expression or function are involved in brain tumorigenesis such as gliomas, neuroectodermal tumours, and neuroblastomas [39].

## 4. Role of IGF-1 in TBI: Experimental Studies 

**Figure 1:**
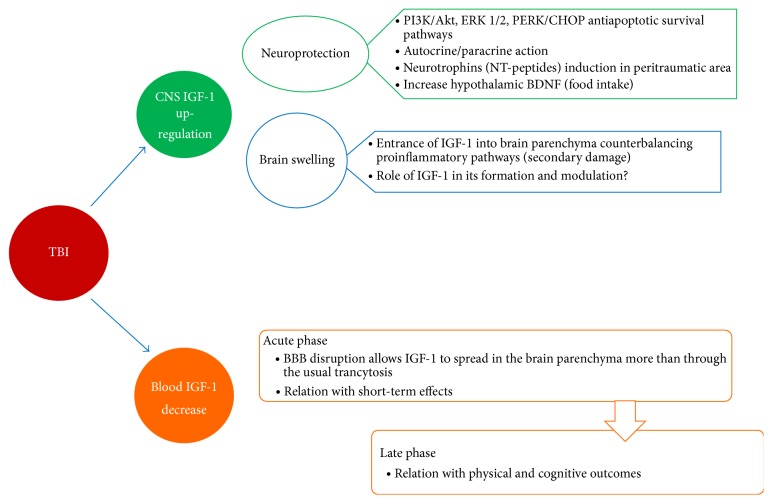
Effects of TBI on IGF-1 expression and metabolism with consequent biological and clinical manifestations.

Apart from its aforementioned role in hypoxic/ischemic stroke, neoplastic and other degenerative diseases, the activity of IGF-1 in the CNS seems to be pivotal even in traumatic brain injuries (Figure 1) with a number of recent findings supporting a role for IGF-1 in wound healing in the brain. IGF-1 is a potent mitogen and can induce differentiation of neural cells in vitro, including neurons, astrocytes, oligodendrocytes, and endothelial cells. It may also influence similar functions in vivo, exerting its mitogenic and trophic effects on a variety of cell types, after brain injury [40], thus leading some authors to study its changes in brain tissue reproducing TBI on animal models [29].

A significantly increased IGF-1 expression after TBI has been widely observed. Li et al. [30] determined the responsiveness of the IGF-1 gene in adolescent mice brain tissue, after penetrating injury; the hormone value was higher on 3 days after injury and remained elevated during the week after, compared to the control group. Sandberg Nordqvista et al. [31] noted an increase of IGF-1 mRNA, with a peak at 24 h after the impact, in their rats contusion model. Madathil et al. [23] also showed a very early (6 h) concomitant increase of IGF-1 in the central area of impact site with a decrease in the injury periphery, where instead IGF-1 hyperexpression was delayed. Walter et al. [32] showed that IGF-1, after penetrating CNS injury in rats, acts in an autocrine/paracrine way to regulate cellular responses, with its limited availability being modulated by the differential presence of stimulatory and inhibitory IGF binding proteins. Several evidences suggest that IGF-1 may play a role in the regulation of reactive astrogliosis, which is one of the most prominent manifestations of the repair response in the mature CNS [41, 42], typically occurring in a delayed fashion within and around areas of neuronal damage, with glial scar formation progressing over several days [43]. IGF-1 has also been proved to stimulate in vitro the astrocyte migration in response to axonal injury [44].

The activities performed by IGF-1 in response to injury begin by binding to its receptor (IGF-1R), which is expressed by neurons, stem cells, and most glial cells [15, 45]. Little is known about IGF-1R expression in response to TBI and Sandberg Nordqvista et al. [31] observed no change in IGF-1R mRNA from 1 to 7 days, following weight drop injury in rats. Instead Walter et al. [32] showed an increased expression of IGF-R protein in the early stage (1–7 days) of penetrant cerebral wounds model. Rubovitch et al. [46] proved that IGF-1R was phosphorylated after mild-TBI, with a time dependent activation at maximum 24 hours. The link between IGF-1 and its receptor leads to the activation of antiapoptotic pathways, whose major are represented by PI3-kinase/Akt and MAP-kinase [47]. As a matter of fact, Madathil et al. [23] showed that, in mouse contusive brain, injury-induced IGF-1 increase may provoke cellular changes through the Akt pathway, as it increases as phosphorylated Akt and/or total Akt, promoting cell survival. Rubovitch et al. [46] confirmed the activation of the Akt pathway and also showed the activation of ERK1/2 following mild-TBI. IGF-1 may even exert its neuroprotective activity after mild-TBI in mice through the PERK/CHOP pathway, which activates the survival/antiapoptotic arm of the endoplasmic reticulum (ER) stress machinery [48].

An interesting role seems to be played by the IGF binding proteins in mediating the activity of IGF-1 after neuronal injury. Usually they are expressed in a variety of tissues and bind IGF-1 and IGF-2, modulating the biological effects with both inhibitory and stimulatory effects [49]. Ni et al. [50] showed that the overexpression of IGFBP-1 impairs brain development and reduces glial cell proliferation in response to injury, in transgenic mice. Sandberg Nordqvista [31] noticed a significant upregulation of IGFBP-2 mRNA in cortical areas close to the injury site and observed a spatial correlation between posttraumatic swelling and increase in IGFBP-2 and -4 mRNA levels. Therefore they hypothesized the involvement of IGF-1 and its binding proteins in the oedema formation and modulation. Walter et al. [32] verified in the acute phase of injury (1–7 days) increased levels of IGFBP-1, -2, -3, -6 localized in injury responsive astrocytes, neurons, and cells of the monocyte lineage, probably facilitating the effects of IGF-1. On the other hand they found a later increase (7–14 days) of IGFBP-4 and -5 localized in the astrocytes and neurons, probably having a role in downregulating the chronic effects of IGF-1. Sandberg Nordqvist et al. [51] also proved that the upregulation of the IGF-1 and IGFBP-2 and -4 is glutamate dependent. Indeed the induction of IGF-1 expression was completely blocked by noncompetitive N-methyl-D-aspartate (NMDA) antagonist (MK-801 or CNQX) in the brain of rats.

The main clinical signs and symptoms reported in patients with mild-TBI include memory disorders and affective lability [52–54]. Many experimental studies suggest that circulating IGF-1 levels are related to cognitive deficits in the aging and amnesic models [55]. The severity of the trauma-induced apoptotic neurodegeneration in the brains of 3–30-day-old rats had been demonstrated to be age dependent and highest in 7-day-old animals. Thus, apoptotic neurodegeneration has been suggested to contribute in an age-dependent fashion to neuropathological outcome of head trauma [56]. Recent results have shown that IGF-1 may even regulate neurogenesis in the adult rat hippocampus [56]. The cognitive dysfunction after TBI may therefore result from hippocampal damage; indeed Schober et al. [57] reported for the first time that hippocampal IGF-1B mRNA increased after developmental TBI in the brain of the rats. Ozdemir et al. [55] proved that the decrease of circulating IGF-1 levels after TBI was associated with cognition and hippocampal damage in 7-day-old rat pups subjected to contusion injury. IGF-1 could also be involved with posttraumatic anxiety disorder. Baykara et al. [58] investigated the effects of progesterone on traumatic brain injury-induced anxiety in 7-day-old rat pups subjected to contusion injury; they found that progesterone treatment decreased TBI-induced anxiety and serum corticosterone levels, while increasing serum IGF-1 levels. In the study of Madathil et al. [43] moderate or severe contusion brain injuries were induced in mice with conditional (postnatal) overexpression of IGF-1, revealing that the astrocyte-derived IGF-1 exerts autocrine effects on astrocytes, reduces regional hippocampal neurodegeneration, and improves posttraumatic cognitive and motor function.

Considering the role of IGF-1 in repair processes, neurogenesis, and posttraumatic anxiety disorders, some authors have conducted experimental studies on the administration of IGF-1.

Assuming the neuroprotective effect of IGF-1 administration in models of cerebral ischemia and spinal cord injury [59], the disruption of blood-brain barrier that starts as early as minutes after brain damage and persists until 7 days after injury, depending on trauma severity [60, 61], may allow systemic IGF-1 to permeate the brain parenchyma improving behavioral outcome in TBI [62]. Based on these evidences, strategies to either increase the endogenous upregulation or supplement it with exogenous IGF-1 may improve neuronal survival after TBI. Kazanis et al. [63], using a model of penetrating brain injury, analysed the beneficial effects of postinjury administration of IGF-1 both at the cellular level and on the animals physical condition. IGF-1 administration resulted in a significant decrease, in the peritraumatic area, of the number of Hsp70 and TUNEL positive cells, which are both typical markers of cell injury. Additionally, they noted an improvement of the total “motor activity” of injured rats, an increased food intake, and an attenuated postinjury body weight loss. In another study Kazanis et al. [40] showed that administering IGF-1 immediately after the trauma reversed the injury-induced decrease in brain-derived neurotrophic factor (BDNF) and neurotrophin-3 (NT-3) in the peritraumatic area, at 4 and 12 h and one week after injury, and it completely voided the effects of injury in the adjacent region. These results demonstrated that IGF-1 administration following TBI could mediate repair and protective processes, also changing neurotrophins levels. Schober et al. [57] found that cognitive outcome improved after administration of erythropoietin (EPO) or insulin-like growth factor-1 (IGF-1), using a controlled cortical impact model of 17-day-old rats. Rubovitch et al. [46] assessed that IGF-1 administration prevented spatial memory deficits following mild-TBI. They also interestingly noticed that blocking the IGF-1R signalling in mild-TBI mice did not increase the spatial memory deficit. The data imply the possibility that the nature of the intrinsic mild-TBI-induced activation of the IGF-1R pathway is different from the one activated by the exogenous administration of IGF-1.

## 5. Role of GH and IGF-1 in TBI: Clinical Studies 

One of the most important consequences of the TBI is the posttraumatic neuroendocrine dysfunction (NED) that refers to a variety of conditions caused by imbalances in the body's hormone production directly related to the pituitary, hypothalamus, and their axes [64]. A recent literature review suggests that the incidence of NED in mild-TBI is 16.8%, while the incidence with moderate TBI has been reported at 10.9% [65]. NED symptoms include fatigue, insomnia, impaired cognition, memory loss, concentrating difficulty, and emotional and mood disturbances, all depending on the severity of the specific hormonal deficiency [66, 67].

The NED pathophysiology following TBI is not completely understood and several mechanisms of injury have been suggested to be involved [68]: compression of the pituitary gland and/or the hypothalamic nuclei due to oedema, skull base fracture, haemorrhage, increased ICP, hypoxic insult, or direct mechanical injury to the hypothalamus, pituitary stalk, or the pituitary gland [69–71]. Nevertheless the factors predisposing to the development of posttraumatic hypopituitarism are still under debate. Some authors postulated that endocrine derangements are related to the severity of the head trauma, as represented by GCS on admission in the ICU, and to high intracranial pressure [72–74]. They demonstrated an association with the extent of brain CT findings [75]; others instead did not find any correlation between the head trauma and the endocrine dysfunction [76, 77]. Overall it seems that the severity of TBI, assessed by initial GCS, is not generally associated with the presence of hypopituitarism, because the initial GCS is not enough discriminative to assess reliably the severity of injury. However a more severe clinical status seems to predict a higher risk of secondary hypogonadism [78]. Therefore routine screening for hormone disturbances in unselected patients after TBI is unlikely to be cost-effective. Screening should be advised in all patients with symptoms and signs of hypopituitarism and a history of TBI and based on earlier reports, probably also in patients with more severe forms of TBI necessitating neurosurgical intervention or admission to an ICU [79]. In both moderate and severe TBI the most affected axis of posttraumatic endocrine dysfunction is the somatotropic one with both cerebrospinal fluid and serum levels of IGF-1 demonstrated to be decreased in adult patients with major head injury [55]. IGF-1 plasma concentrations in patients with TBI are typically below the normal physiologic range of 150–400 ng/mL [80]. In literature GHD prevalence varied from 2 to 66%, with up to 39% of cases suffering from severe deficiency [81]. Several factors could explain this percentage variability, including different time interval between TBI and the assessment of pituitary function (from 24 h to 35 years), type and severity of the brain injury, different methods to evaluate pituitary function reserve such as tests and hormonal assays, criteria for the diagnosis, and selection criteria not excluding those patients in whom, besides a history of TBI, alternative causes of pituitary dysfunction have not been ruled out [82, 83]. To avoid this bias, patients should be followed up at least 1 year after the trauma, as suggested in the consensus guidelines for the evaluation and diagnosis of patients with possible GHD [83]. The absence of a gold standard test for GHD will always raise questions regarding the true occurrence of a GH deficiency after TBI [84]. Therefore analyses of GH especially under multiple pharmacological treatment, as in TBI patients, should be interpreted with caution [85]. Consensus guidelines to overcome confounding factors in TBI patients state that the GH/IGF-1 deficiency should be evaluated through a first line measurement of the basal anterior pituitary hormones, by dynamic endocrine testing such as the glucagon stimulation test, followed by the second line growth hormone releasing tests (GHRH), arginine test and GHRH + GHRP-6 and/or insulin tolerance test [86, 87]. Moreover, the results of GH stimulation tests are confounded by BMI, with higher BMI being associated with decreased GH responses. Although BMI-adjusted reference values have been reported, none of the studies on TBI-associated GHD reports adjusted their cut-off values for BMI [83].

It seems fairly accepted that in the long-term phase of TBI (3 months onwards) GH and IGF-1 blood levels appear frequently reduced with different prevalences. In this sense Kelly et al. [70] found 18% GH defect among patients with TBI, and Lieberman et al. [76] reported 15%, whereas Agha et al. [88] and Aimaretti et al. [89], respectively, indicated 18% and 37% of GH reduction and Abadi et al. [90] showed IGF-1 deficiency in 24% patients three months after injury. These controversial data are certainly due to the lack of standardization of the patients cohorts, the inclusion of different types of severity of the trauma (mild, moderate, and severe), and the different methods used in the hormonal dosage.

A contradictory literature characterizes even the discussion about GH and IGF-1 levels following TBI in the acute phase of injury. Plasma IGF-1 concentrations do not seem to be a reliable reflection of GH secretion or action in the setting of acute illness [88]. In fact some authors reported an increase of GH levels in the acute phase and others show a relation with high ICP [72, 91]. Other studies instead show GH levels remaining relatively normal or slightly elevated throughout the acute setting in mild, moderate, and severe TBI [92, 93].

On the other hand some evidences suggest that IGF-1 decrease in the acute phase of injury with reduced serum IGF-1 and IGFBP-3 levels reported in the first 48–60 h following TBI [94]. In a recent study [85] a transient decrease in serum IGF-1 has been recognized with low levels on day 1 and then restored towards normal on day 4 after severe TBI. Interestingly blood IGF-1 levels do not appear to be related to GH value in the acute phase of injury. In fact low IGF-1 with elevated GH levels have been shown in the acute posttraumatic phase, as well as a normalization of GH with an increase of IGF-1 in the following weeks after the acute event [95]. According to these data Agha et al. and Dimopoulou et al. showed no statistical differences in plasma IGF-1 concentrations between the GH-sufficient and GH-deficient groups, after severe TBI [75, 88].

The detection of a peripheral resistance to GH action, manifested by elevated plasma GH concentrations, with low plasma IGF-1 concentrations, underlines the influence on plasma IGF-1 levels even by factors other than GH secretion and action [88, 96]. Although GH and nutrition represent the major factors regulating IGF-1 expression in the liver, as well as in a number of other organs [97], in some tissues IGF-1 expression appears to be modulated by specific trophic factors. In this sense there are evidences supporting injuries as factors able to influence the brain expression of IGF-1 [39] as much as GH [98] and nutrition [99] do. The role of the trauma-induced elevation in IGF-1 is unclear, but it is feasible that IGF-1 upregulation in surviving neurons may act to limit the progression of cell death, induce progenitor cell differentiation, or promote neurite outgrowth [23].

## 6. GH and IGF-1 Deficiency

Several pieces of data clearly demonstrated that GH deficiency is the most common pituitary deficit with a 20% incidence of severe GHD one year after TBI. In patients with mild and moderate traumatic brain injury, pituitary function may improve over time in a considerable number of patients but, although rarely, may also worsen over a 3-year period. Patients with severe TBI, instead, usually suffer from persistent GHD up to 3 years after trauma [100]. Normal pituitary function in the short term, although rarely, becomes impaired later on. Thus, brain-injured patients must always undergo neuroendocrine follow-up over time to monitor pituitary function and eventually provide appropriate hormonal replacement [67].

It is widely accepted that the somatotropic axis plays both a central role in the development and growth of CNS and a protective role in dementia, traumatic and ischemic injuries of the brain [101]. The major studies used the GHRH-arginine test as the primary test to evaluate the GH-IGF-1 axis, adopting a peak GH of 9.0 mcg/L as a cut-off value, whereas recent clinical practice guidelines recommend a limit value of 4.1 mcg/L [102]. According to a multicenter study, which used a sensitive immunochemiluminescent two-site assay, this cut-point provides the best compromise in terms of specificity and sensitivity for the diagnosis of adult GH deficiency through the GHRH-arginine test, thus minimizing the misclassification of multiple pituitary hormone deficiencies and control subjects [103].

GH and IGF-1 deficiencies are associated with multiple physical, metabolic, and neuropsychological manifestations including diminished lean body mass, disrupted lipoprotein and carbohydrate metabolism, reduced bone mineral density, and impaired cardiac function, as well as decline in cognitive functioning, fatigue, and diminished quality of life [104].

In the early stages of life, growth retardation after TBI is the hallmark of potential damage to the hypothalamic-pituitary function of the GH/IGF-1 axis. Because of the similarity of some TBI sequelae to those of untreated hypopituitarism, it is frequently postulated that hormone deficits may contribute to the chronic disability of TBI survivors. In this context, a recent study has shown that GH-insufficient TBI patients have higher levels of fatigue than GH-sufficient TBI patients up to 6–9 months after the trauma [105]. Thus, providing appropriate diagnosis of this deficiency is crucial, as the subsequent management using growth hormone (GH) replacement therapy has been ascertained to be effective [106].

It has also been noticed that the combination of IGF-1 and GH therapy improves metabolic and nutritional parameters after TBI. IGF-1 induced changes of BDNF in the anterolateral hypothalamic area could be related to the effects of IGF-1 in controlling food intake, which have been implicated in the protective actions of IGF-1 following injury, since BDNF levels have been shown to change in conditions of altered food intake [40]. More specifically the ameliorative effect of IGF-1 could be primarily attributed to its effect in increasing food intake, the parameter shown to have the strongest improvement since inadequate nutrition is known to be a major clinical problem following brain trauma. The latter typically causes a hypermetabolic stress to the organism and IGF-1 is shown to act as a potent anabolic agent in such cases [64].

A postinjury rapid increase in plasma IGF-1 concentrations to more than 350 ng/mL seems transiently to improve both nitrogen retention and trend in 6-month outcomes [107]. Indeed Hatton et al. [108], comparing combination IGF-1/GH therapy and a placebo treatment on 97 patients with moderate to severe TBI, noticed a positive nitrogen balance during the first 24 hours in the treated group with a positive trend throughout all the treatment period. The combination of IGF-1 and GH after moderate to severe acute TBI produced sustained improvement in metabolic and nutritional end-points, such as the hyperglycemia, insulin resistance, and compromise of the immune system and continous loss of protein.

## 7. Discussion

Worldwide TBI is one of the major causes of death and disability. This is why understanding how to prevent secondary damage and improve outcomes in patients suffering from head injury has always been a target of scientific interest. An increasing number of experimental results suggest that most of the long-term consequences of TBI are due to molecular and cellular changes that occur during the acute phase of the injury and which persist, or even progress, subsequently [40]. Thus nowadays, the success of therapeutic interventions following TBI is strongly dependent even on the complex molecular signalling cascades targeting [9].

Many authors focused their attention on the posttraumatic neuroendocrine dysfunction in order to achieve a correlation between hormones blood level and TBI outcomes.

In the contemporary literature the hormonal processes belonging to the somatotropic axis result to be the most affected by TBI, with different alterations between the acute and late phases. Levels of IGF-1 transcript begin to increase between 1 and 3 days after lesion and remain elevated throughout the second week following injury. These results further support a role that locally produced IGF-1 is the expression of the brain's response to injury [30]. Specific studies could not, however, determine whether the increased concentration of IGF-1 resulted from local synthesis within the damaged region or from damaged blood vessels since serum has the highest levels of IGF-1 in the body [64]. Nevertheless the specific IGF-1 upregulation at the site of the lesion has led to the suggestion that IGF-1 may be involved in the process of tissue healing, playing a role in the neuroprotective and/or neurorepairing response of brain tissue to trauma [40].

In the long-term evaluation the serum levels of GH and IGF-1 seem to decrease, determining multiple physical, metabolic, and neuropsychological manifestations. Their early recognition and prompt replacement therapy are likely to be crucial in the management of GHD patients recovering from TBI [84]: authors adopting a combination therapy of GH and IGF-1 showed improved outcomes, taking in account both physical and cognitive aspects.

Therefore an important question is whether circulating IGF-1 levels are predictive of cognitive dysfunction resulting from hippocampal damage following traumatic injury especially in developing brain. In animal models, it was shown that decreased serum IGF-1 levels resulted in cognitive deficits and IGF-1 deficiency led to impaired learning and memory in adulthood. Various experimental studies found that low-serum IGF-1 levels were related to cognitive dysfunction following traumatic injury. Further studies need to be carried out on human subjects or experimental models in order to evaluate the time course or damage-dependent IGF-1 levels in TBI. Therapy strategies that increase circulating IGF-1 may be highly promising, in this sense, for preventing the unfavorable outcomes of traumatic damage particularly in young children [55].

It is still controversial whether an alteration of blood IGF-1 is due to its subordination to GH or not. Moreover some studies have recently demonstrated that the evaluation of neuroendocrine processes in the acute phase of the injury also involves a peripheral resistance to GH actions, thus highlighting other factors likely influencing IGF-1 levels.

Although a recent study shows no correlations between IGF-1 levels and 3 months' outcome [85], experimental studies in animals have revealed a role of IGF-1 in the context of the secondary mechanisms of response to posttraumatic damage. In fact many authors verified that while the systemic level of IGF-1 decreases, the CNS responds to the injury upregulating the expression of IGF-1. A subset of molecules in the IGF cascade thus responds to traumatic injury with transient but striking increase in mRNA synthesis. It is possible that the selective change in IGF binding protein mRNAs seen following injury serves to relocate growth factors to cells in need of posttraumatic repair [23, 30, 31]. Therefore IGF-1 behaves as a neuroprotective peptide, activating many signalling pathways that promote cells survival, acting in an autocrine/paracrine way to regulate cellular responses, regulating the reactive astrogliosis while stimulating proliferation and differentiation of oligodendrocytes that support myelination of the CNS. Astroglial cells provide physical and metabolic support for neurons and their processes often end on blood vessels. They are highly enriched in IGF-1 receptors and IGF-1 has stimulatory effects on astrocyte multiplication and glucose uptake [51]. Astrocytosis may also be beneficial after injury by forming a physical and biochemical barrier to separate a contused area from healthy tissue, limiting the spread of inflammatory molecules and cells. Indeed, removal of reactive astrocytes after TBI has been shown to worsen tissue loss and behavioral performance [43].

Numerous experimental studies have shown that IGF-1 provides long-term protection to mature oligodendrocytes, mainly by inhibiting oligodendroglial apoptosis but also through its mitogenic properties upon the oligodendroglial precursors. Irrespective of the mechanisms, IGF-1 induced maintenance of neurotrophins within the peritraumatic area could be involved in the previously reported effect of IGF-1 in preserving tissue homeostasis [40].

An interesting role seems to be played by IGF-1 in the development of posttraumatic oedema, which represents a critical and therapeutically insidious problem in TBI patients, especially in moderate and severe injuries. It is possible that localized breakdown of the blood-brain barrier could increase local brain levels of selective growth factors and thus contribute to the wound healing process. Glial cells have been reported to internalize plasma proteins and retain them over a long term, suggesting that extravasated plasma proteins may serve physiological functions in wound healing [109].

In the eventuality that IGF binding proteins are involved in the pathogenesis of oedema formation, modulation of the molecular components in this response may be an accessible route towards the development of novel therapeutic agents aimed at minimizing brain damage [31]. Although it is not well ascertained if these proteins belong to the proinflammatory pathway of oedema or rather if they play a protective anti-inflammatory role, certainly this is a root to figure out if by rating the expression of IGF and its binding proteins the time of posttraumatic oedema formation and all its sequelae could be monitored. Since central administration of IGF-1 can rescue neurons in the cortex, striatum, hippocampus, dentate gyrus, and thalamus following hypoxic injury, local production following injury is believed to be meaningful in the wound healing process [109]. Knowledge of the stepwise events controlling wound healing following brain injury should contribute to the tailored treatment of affected patients.

Administration of peptides or drugs which induce or repress peptide expression may optimize healing and minimize excessive scar tissue formation. As gene therapy techniques become increasingly sophisticated and efficient, expression plasmid, viral vector, or oligonucleotide administration may become commonplace strategies. Treatments may be most effective if tailored to specific forms of injury or to specific regions of the brain. Nevertheless, effective treatment of brain injuries with drugs, peptides, or genes will require a thorough understanding of the complex cellular changes and intercellular interactions which occur following the insult [108].

## 8. Conclusions

Strategies to either increase the endogenous upregulation of IGF-1 after TBI or supplement it with exogenous IGF-1 may improve neuronal survival after TBI [23]. In this context, the use of antidiabetic agents (e.g., metformin) and GLP-1 mimetic agents (e.g., liraglutide) has been suggested. These drugs cross the BBB, elicit neuroprotective activities, and, importantly, are safe and well-tolerated medicines. Along with more recent data linking brain insulin/IGF-1 function to the etiology of a number of neurodegenerative diseases will, undoubtedly, translate into more clinically oriented avenues of research in the near future. Depending on each personal genetic background, antidiabetic drugs and other molecules potentially interacting with the IGF-1 system may probably play a role in the next future when facing TBI and other nervous system pathologies. It is expected that future studies will take advantage of postgenomic technologies in order to generate molecular and/or biochemical signatures aimed at identifying patients who may benefit from these therapies [12].

We believe that new prospective studies should investigate the changes of IGF-1 in blood and assess their possible correlation with the cascade of events secondary to trauma.

The identification of IGF-1 as a biomarker of posttraumatic injury could help in the future to understand whether and how to plan the hormone replacement therapy to prevent secondary damage of trauma and to improve the patients outcome.

It is too early to figure out IGF-1 as a strategic agent in a therapeutic context. Moreover in terms of drugs and other therapies, research suggests that a single highly effective pharmacological agent for TBI is still unlikely to be discovered but that improved knowledge of the pathophysiology, together with the continuing advances in the field of gene therapy, will provide the mechanistic clues to direct a mosaic of therapeutic interventions.

## Abbreviations

CNS: Central nervous system
TBI: Traumatic brain injury
CT: Computed tomography
MRI: Magnetic resonance imaging
CSF: Cerebrospinal fluid
GH: Growth hormone
IGF: Insulin-like growth factor
IGFBP: Insulin-like growth factor binding protein
BBB: Blood-brain barrier
AD: Alzheimer's disease
NED: Neuroendocrine dysfunction
GHD: GH deficiency
BDNF: Brain-derived neurotrophic factor.
